# Supplementary material for: Influence of COVID-19 confinement on students’ performance in higher education
Source: PLoS One. 2020 Oct 9;15(10):e0239490. doi: 10.1371/journal.pone.0239490 (PMC7546684; doi:10.1371/journal.pone.0239490)
Supplement: Attachment — Submitted filename: ListOfChanges.docx [file pone.0239490.s002.docx]

**Dear editor,**

**we have carefully revised all the comments made by the reviewers and we have modified the manuscript accordingly. Answer to all the points can be found below. We have made additional changes that are also indicated at the end of this document.**

**Reviewer 1:**

The organization of the results section and the addressed questions (1-4) is a bit unclear please reorganize this so it becomes clear what analysis has been used to address what question.

We have included explicit references to the analysis that has been used to answer each research question. We have also clarified the point of the discussion that jumps from answering research question 1 to research question 2, which was the most confusing part of the discussion.

Abstract:
This study explores the effects of COVID-19 confinement in the students’ performance in higher education. This sentence should be rephrased more clearly without genitive form.

That sentence has been rephrased as reviewer suggested.

The language of the whole paper should carefully revised. At the moment the paper is hard to understand.

We have carefully revised the language in the whole paper.

In this subject we have analyzed students’ performance expressed... I needed to guess that the name of the subject is 'applied computing'. This needs to be expressed more clearly, also in the section headings: For instance:
2.3.1 Subject: Applied computing

We have changed the order of the information in section 2. Now, the description of the subjects is placed before the statistical analysis. In the subsection participants, we have also included a sentence that explicitly indicates the name of the subjects, that are described right after that sentence.

Question 2: The results of our studies clearly indicate that there are significant differences in students’
519 performance after the confinement that cannot be found before in the same year or when comparing to the previous
520 academic years. It is unclear how this has been established statistically. Please clarify.

The reviewer is right when asking about clarification in this sentence because the sentence was not correctly written. In the present version, it reads:

“The results of our studies (section 3.2) clearly indicate that there are significant differences when comparing students’ performance in confinement with the performance in previous periods where activities were not limited to distant learning.”

This is one of the main results of the article and it has been established statistically in section 3.2

Where is the Wilcox test for Fig 7 comparing the academic years?

Data from this subject imply results from an adaptive test. This imply that there are some points in the test where students get stacked and some scores are repeated more often than others. For this reason, data did not pass the normality test, as we explain in section 2.4.1. We have used non-parametric statistical tests such as Kruskal-Wallis and Mann-Whitney post hoc test, as explained in the same section. Results including the statistical analysis are included in the third paragraph of section 3.2.1. (starting in line 452).

We have extended the explanation in section 2.4.1.

I missed how the following has been studied: How do you exclude the possibility that the lecturers just gave better scores? Given the fact that the university would not like to see worse grade of the students because this could reflect negatively on the institution and their management this is a valid question. Please address it.

Thank you for asking that important question, that must be clarified in the article indeed. The subjects involved in this article mainly include tests that are objective, such as multiple answer tests or open questions with a very specific and quantitative answer that cannot be altered by the teachers. For that reason, we are sure that it is impossible for lecturers to give better scores.

We have included a point that indicates this fact in the discussion and conclusions section.

**Reviewer 2:**

Aims of the study were clearly defined and followed, data were appropriately collected and analysed, results were adequately discussed, and conclusions were well supported. I propose the following minor revisions.

1.The first part of the “introduction” focuses on the assessment, which is not appropriate given the main purpose of the study is to investigate the influence of COVID-19 confinement. I would recommend to start directly with “1.1 background” with a brief introduction of the e-learning in the situation of COVID-19.

The first part of the introduction, (section 1) talks in general terms about the problem studied in the article. Then, in section 1.1 we give more details about several topics such as w-learning, Self-regulated learning and adaptive tests. For that reason, we do not think it is adequate to completely remove section 1 and start directly in section 1.1. however, reviewer comments are right in the sense that, maybe the part that talks about assessment is too long and an introduction about e-learning in confinement is required.

For those reasons, we have reduced the assessment discussion in section 1.1 and a brief introduction of the e-learning in the situation of COVID-19 has been added in section 1.1 Background.

2.It would be much clearer if the section “participant” is placed before the section of “statistical analysis”.

We have modified the order of these subsections as reviewer suggested.

3.The results presented in figures 4-6 are not reader-friendly.

Figures have been modified to increase readability by increasing the size of the smaller letters. In figure 6, we have changed even the structure of the figure to make it more reader-friendly.

**Additional changes**

1. Changed the word “significative” for “significant” in 5 places
2. Notation changes in the section “2.1.1 CAT theoretical model” (some changes of psi by fi)
3. The affiliation Universitat Oberta de Catalunya has been added to the author Laia Subirats
4. Affiliations have been renumbered by order of appearance
